# Prediction of prodromal symptoms and schizophrenia-spectrum personality disorder traits by positive and negative schizotypy: A 3-year prospective study

**DOI:** 10.1371/journal.pone.0207150

**Published:** 2018-11-08

**Authors:** Anna Racioppi, Tamara Sheinbaum, Georgina M. Gross, Sergi Ballespí, Thomas R. Kwapil, Neus Barrantes-Vidal

**Affiliations:** 1 Departament de Psicologia Clínica i de la Salut, Universitat Autònoma de Barcelona, Barcelona, Spain; 2 Department of Psychology, University of Southern California, Los Angeles, California, United States of America; 3 VA Connecticut Healthcare System, West Haven, Connecticut, United States of America; 4 Yale School of Medicine, New Haven, Connecticut, United States of America; 5 Department of Psychology, University of Illinois at Urbana–Champaign, Champaign, Illinois, United States of America; 6 Sant Pere Claver–Fundació Sanitària, Barcelona, Spain; 7 Centre for Biomedical Research Network on Mental Health (CIBERSAM), Instituto de Salud Carlos III, Barcelona, Spain; Department of Psychiatry and Neuropsychology, Maastricht University Medical Center, NETHERLANDS

## Abstract

The present study extends previous cross-sectional findings by examining the predictive validity of positive and negative schizotypy in a young adult sample at a three-year follow-up. Schizotypy and schizophrenia share a comparable multidimensional structure with positive and negative dimensions being the most strongly supported factors. Previous cross-sectional and longitudinal studies employing the psychometric high-risk strategy indicated that schizotypy is a useful method for identifying risk and resilience factors for the development of schizophrenia-spectrum psychopathology. In the present study, 103 participants (77% of 134 candidate participants) were reassessed at a three-year follow-up. As hypothesized, positive schizotypy predicted psychotic-like symptoms, depression, low self-esteem, and general psychopathology. Negative schizotypy predicted emotional disturbances, schizoid personality traits, and mental health treatment during the past year. As expected, both schizotypy dimensions predicted schizotypal, paranoid, and avoidant personality traits, and impaired functioning. These longitudinal findings provide additional evidence supporting the multidimensional model of schizotypy as a valid framework for studying etiological mechanisms and trajectories of psychosis.

## Introduction

Schizotypy is operationalized as a continuum of subclinical and clinical symptoms, and impairment that in the extreme is manifested as schizophrenia-spectrum disorders [1, 2]. This construct emerged from two different traditions: clinical psychopathology and individual differences [1, 3]. The former observed that there were mild forms of schizophrenia-like features in relatives of affected persons and ambulatory patients, which led to the conceptualization of schizotypy as a soft version of schizophrenia psychopathology. From this viewpoint, schizotypy is represented as personality pathology positioned at the beginning of the disease process, and dimensionality is thought to exist as a degree of clinical severity. From the personality tradition, schizotypy is conceived as both variation in healthy personality and as a risk factor for psychosis.

Schizotypy offers a unifying framework that encompasses subclinical expressions, the psychosis prodrome, schizophrenia-spectrum personality disorders, and psychotic disorders. Recent conceptualizations consider schizotypy as a distal risk marker for the identification of individuals at risk for schizophrenia, as well as a developmental mediator along the risk trajectory of schizophrenia-spectrum disorders [4]. From this perspective, schizotypy allows us to advance our understanding of etiological factors (including risk and protective factors) for schizophrenia and spectrum disorders without the confounds related to such illnesses [5, 6].

Schizotypy, like schizophrenia, is heterogeneous in terms of etiology and expression, and this heterogeneity can be captured by a multidimensional structure, with positive and negative schizotypy being the most widely replicated factors [2, 7–9]. Positive schizotypy is characterized by odd beliefs, unusual perceptual experiences, and suspiciousness, whereas negative schizotypy involves diminished functioning such as anhedonia, affective flattening, and social disinterest. Overall, positive and negative schizotypy dimensions demonstrate solid construct, concurrent, and predictive validity in psychometric high-risk studies [10–12], cross-sectional interview and questionnaire studies (e.g., [13–17]), and in daily life studies using experience sampling methodology (ESM) [18–20]. These studies indicate that positive and negative schizotypy present certain commonalities and that are also differentially associated with psychopathology, personality, and impairment. Cross-sectional interview studies [13, 15, 17] have commonly reported that positive schizotypy is associated with positive (psychotic-like) symptoms, mood disorders, and substance abuse, whereas negative schizotypy is associated with negative and schizoid symptoms. Furthermore, both positive and negative schizotypy are associated with schizotypal and paranoid symptoms, and with impairment in general and social functioning. Additionally, studies conducted in the domain of daily-life with ESM expand and add ecological validity to the above mentioned findings. This work has demonstrated that positive schizotypy is associated with increased stress reactivity and negative affect in the moment, as well as with psychotic-like experiences and suspiciousness, whereas negative schizotypy is associated with diminished social contact in daily-life and emotional reactivity, as well as with negative symptoms and decreased positive affect in the moment [18–20]. Specifically, Kwapil et al. [18] reported that both schizotypy dimensions were associated with the desire for solitude when with others. However, in individuals high on positive schizotypy the preference for solitude was moderated by anxiety symptoms, whereas in individuals high on negative schizotypy the association was moderated by decreased positive affect. Barrantes-Vidal et al. [19] showed that stress in the moment was associated with experiencing psychotic-like and paranoid symptoms and also predicted psychotic-like symptoms at the subsequent moment, but only for individuals with high positive, but not negative, schizotypy.

Debbané and colleagues [21] identified only six longitudinal studies investigating schizotypy in general population samples (and three of those studies drew from the same sample). These studies indicated that schizotypy dimensions are differently associated with the development of schizophrenia-spectrum symptoms and disorders, and reinforce the validity of schizotypy as a useful multidimensional construct for identifying individuals at-risk. The classic study of Chapman et al. [22] examined 534 college students identified by the Wisconsin Schizotypy Scales (WSS), including the Perceptual Aberration (PerAb) [23], Magical Ideation (MagicId) [24], and the Physical Anhedonia (PhyAnh) [25] scales. They successfully reinterviewed 508 subjects at a ten-year reassessment, reporting that participants identified by the PerAb and MagicId Scales (measures of positive schizotypy) had elevated rates of psychotic disorders and schizophrenia-spectrum symptoms at the follow-up. Using the Chapmans’ longitudinal data, Kwapil [26] reported that high scores on the Revised Social Anhedonia Scale (SocAnh) [27] had elevated rates of schizophrenia-spectrum disorders and symptoms at the ten-year follow-up. In a further reanalysis of the Chapman et al. [22] data, Kwapil et al. [11] computed dimensional positive and negative schizotypy scores based on the WSS. Positive schizotypy predicted the development of psychotic disorders, whereas both positive and negative schizotypy predicted schizophrenia-spectrum disorders. Gooding et al. [28] conducted an independent psychometric high-risk study with a five-year follow-up period of participants identified by the PerAb, MagicId, and SocAnh Scales. They reported that the SocAnh group had elevated rates of schizophrenia-spectrum disorder diagnoses compared to both the PerAb/MagicId and control groups. However, in contrast to Chapman et al. [22] findings, none of the participants met diagnostic criteria for a psychotic disorder at follow-up. Gooding and colleagues [28] argued that this discrepancy probably reflect the differences of the proportion of lifetime risk for psychosis covered by their 5-year follow-up compared to the Chapman’s 10-year follow-up. However, consistent with Kwapil [26], they indicated that SocAnh identified individuals at specific high risk for the development of schizophrenia-spectrum disorders.

In 2011, Barrantes-Vidal and colleagues (e.g., [15]) began a new longitudinal study of college students assessed for positive and negative schizotypy. Consistent with previous studies, their initial interview assessment (completed 1.7 years after the schizotypy screenings) supported the construct validity of both schizotypy dimensions. Positive and negative schizotypy dimensions were associated with schizotypal and avoidant personality traits, suspiciousness, and impaired functioning. Negative schizotypy was associated with negative symptoms and schizoid personality. Positive schizotypy was associated with psychotic-like experiences, negative affect, and borderline and paranoid personality traits. In addition, both dimensions demonstrated differential associations with cognitive schemas. Positive schizotypy was associated with elevated negative interpersonal schemas, whereas negative schizotypy was associated with diminished positive views of self and others.

The extant cross-sectional and longitudinal findings are striking in that nonclinically ascertained participants demonstrate comparable patterns of symptoms and impairment (albeit at a milder level) as patients with schizophrenia-spectrum disorders. Furthermore, nonclinically ascertained young adults who endorse schizotypic traits are at elevated risk for the development of schizophrenia-spectrum symptoms and disorders. However, such longitudinal studies are rare, and only one [11] examined the predictive validity of psychometrically identified positive and negative schizotypy dimensions. The present work employs a prospective framework to study the expression of positive and negative schizotypy in a young, general population sample and assesses the construct validity of multidimensional schizotypy as an indicator of schizophrenia-spectrum psychopathology.

### Goals and hypotheses

The present study further examined the validity of psychometrically assessed positive and negative schizotypy in a nonclinically ascertained sample of young adults at a three-year follow-up assessment (Time 3; T3) of the sample initially reported by Barrantes-Vidal et al. [15]. The first goal of this study was to extend our previous findings by examining, in a longitudinal framework, whether baseline (Time 1; T1) positive and negative schizotypy ratings differentially predicted symptoms and functioning at the three-year reassessment. It was hypothesized that both positive and negative schizotypy dimensions would be associated with schizotypal, paranoid, and avoidant personality traits, and impaired functioning, but that positive schizotypy would be specifically associated with positive (psychotic-like) symptoms, depression, and low self-esteem, whereas negative schizotypy would specifically predict negative symptoms, schizoid personality traits, and emotional blunting. Secondly, we examined whether reports at the Time 2 (T2) interview assessment (1.7 years after T1 and 1.4 years before the current T3 assessment) of symptoms and impairment predicted the same constructs assessed at T3. It was expected that in general T2 measurements would be predictive of the T3 scores, with symptom measures showing lower stability than trait measures. Finally, we examined whether positive and negative schizotypy assessed at T1 predicted the associations of these constructs (i.e., temporal stability or maintenance of symptoms from T2 to T3). We expected that high levels of baseline schizotypy would predict maintenance of symptoms from T2 to T3, whereas low levels of baseline schizotypy would predict low stability.

## Method

The method employed to select participants is available as supporting information; see Protocol of the Psychometric High-Risk Strategy Project for Examining Risk and Resilience Trajectories across the Psychosis Continuum dx.doi.org/10.17504/protocols.io.ubieske

### Participants and procedure

The present assessment is part of an ongoing longitudinal study examining risk for schizophrenia-spectrum psychopathology. Participants were initially screened and recruited from psychology courses at Universitat Autònoma de Barcelona. As described in Barrantes-Vidal et al. [15], a total of 589 unselected students completed self-report questionnaires at T1, with usable screening data obtained from 547 participants (mean age = 20.6; SD = 4.1; 86% female). In order to have continuous distributions of scores on the schizotypy dimensions with an adequate representation of high scorers, we invited all 189 participants who had standard scores based upon sample norms of at least 1.0 on the positive or negative schizotypy factors from the WSS, the suspiciousness subscale of the Schizotypal Personality Questionnaire (SPQ) [29], or the positive symptom subscale of the Community Assessment of Psychic Experiences (CAPE) [30], and 150 randomly selected participants who had standard scores < 1.0 on each of these measures to participate at T2. Participants were assigned positive and negative schizotypy factor scores based upon norms from 6137 American young adults [17]. Note that Kwapil et al. [31] demonstrated that the positive and negative schizotypy factor structure underlying the scales was invariant in Spanish and American samples. The Spanish adaptation of the WSS used [32] has shown good reliability in college samples as well as external validity (e.g., [33]). Furthermore, the norm-based factor scores correlated .99 with factor scores generated from a principal components analysis with the Spanish sample of 547 participants.

At T2, 214 participants (mean age = 21.4 years; SD = 2.4; 78% female), completed the assessment (described in Barrantes-Vidal et al. [15]). The sample included 123 participants with at least one schizotypy screening score above 1.0 and 91 with standard scores below 1.0. The mean interval between T1 and T2 assessments was 1.7 years (SD = 0.2 years, range 1.4 to 2.2 years).

Due to funding limitations, we selected a sub-sample of the T2 participants that retained a similar distribution of schizotypy scores for assessment at T3. We recruited 134 participants (93 with high schizotypy and 41 with standard scores below 1.0). Of these, 103 (77%) participants (mean age = 23.06; SD = 2.6; 37.9% male) were reassessed, 75 of 93 (82%) participants with elevated schizotypy scores and 28 of 43 (65%) with standard scores below 1.0. There were no significant differences on positive or negative schizotypy scores between the participants assessed at T3 and the non-followed participants. The mean interval between T2 and T3 assessments was 1.4 years (SD = 0.3 years, range 0.9 to 2.1 years) and between T1 and T3 assessments was 3.1 years (SD = 0.3 years, range 2.6 to 3.6 years). At each assessment, participants provided informed consent and ethical approval was granted by the Ethics Committee of the Universitat Autònoma de Barcelona (Comissió d'Ètica en l'Experimentació Animal i Humana (CEEAH); number 701H-JS; http://www.recerca.uab.es/ceeah/).

### Materials

#### Time 1 self-report measures

All 547 participants at T1 were administered the WSS intermixed with an infrequency scale [34] and the CAPE and SPQ-suspiciousness scale. The Wisconsin Schizotypy Scales were used to assess positive and negative schizotypy traits. The Perceptual Aberration Scale [23] assesses psychotic-like bodily distortions and perceptual experiences; the Magical Ideation Scale [24] taps belief in invalid causation; the Revised Social Anhedonia Scale [27] measures schizoid asociality; and the Physical Anhedonia Scale [25] assesses deficits in sensory and esthetic pleasure. The CAPE [30] assesses positive, negative, and depressive dimensions of the psychosis spectrum. The positive dimension scale contains 20 items and was used in this study to assess psychotic-like experiences. The SPQ [29] is a measure of schizotypal personality traits as defined in the Diagnostic and Statistical Manual of Mental Disorders [35]. The 8-item Suspiciousness subscale was used to assess suspiciousness/paranoid ideation.

#### Time 2 and Time 3 self-report and interview measures and procedures

Participants at the T2 and T3 assessments were administered questionnaires and diagnostic interviews (along with measures not reported in this study). The interviews were conducted by psychologists and advanced graduate students in clinical psychology. All interviewers were extensively trained and were unaware of participants' scores on the T1 and T2 measures.

Participants completed the Rosenberg Self-Esteem Scale [36]. The Comprehensive Assessment of At-Risk Mental States (CAARMS) [37] is a structured interview that assesses the psychosis prodrome and is used to assess psychotic-like symptoms in nonpatients. Severity scores for seven CAARMS subscales were used. Interview information collected in the CAARMS was used to rate the Structured Interview for Prodromal Symptoms (SIPS) and the Scale of Prodromal Symptoms (SOPS) [38] positive, negative, disorganized, general and total symptom dimensions. The Structured Clinical Interview for DSM-IV Axis II Disorders [39] was used to assess schizophrenia-spectrum personality disorders and obtain dimensional ratings for paranoid, schizoid, schizotypal and avoidant personality disorders. Functioning was rated using the Social and Occupational Functioning Assessment Scale [40] and the Global Assessment of Functioning [41]. Depressive symptoms were assessed with the Calgary Depression Scale [42] and the Beck Depression Inventory-II [43].

## Results

### Descriptive statistics

The mean for positive schizotypy assessed at T1 was −.05 (SD = 1.07, range = −1.45 to 3.23), and for negative schizotypy was .20 (SD = 1.17, range = −1.57 to 4.27). Both dimensions were unimodal and positively skewed. The schizotypy dimensions were not correlated (r = .03). In addition, the mean for CAPE positive symptoms dimension assessed at T1 was 9.46 (SD = 5.31, range = 0 to 23), and for SPQ suspiciousness subscale was 3.48 (SD = 2.29, range = 0 to 8). Table 1 provides descriptive data for the measures used in the study.

**Table 1:** Descriptive statistics for quantitative dependent measures of symptoms, impairment, and personality.

	Time 2				Time 3			
Measure	Mean	SD	Range	Alpha ^a^	Mean	SD	Range	Alpha ^a^
CAARMS positive symptoms	1.55	2.77	0–16	-	1.21	2.16	0–12	-
CAARMS negative symptoms	1.90	2.72	0–11	-	1.63	2.34	0–9	-
CAARMS cognitive symptoms	1.11	1.80	0–8	-	1.02	1.51	0–7	-
CAARMS emotional disturbance	1.23	2.11	0–8	-	0.91	1.46	0–6	-
CAARMS behavioral symptoms	1.64	2.19	0–8	-	1.71	2.24	0–9	-
CAARMS motor/physical symptoms	1.18	2.15	0–14	-	1.14	1.87	0–10	-
CAARMS general psychopathology	3.85	4.15	0–21	-	4.63	4.33	0–22	-
Schizotypal personality ratings	1.46	2.35	0–13	-	1.33	1.98	0–10	-
Schizoid personality ratings	1.15	1.76	0–8	-	1.01	1.80	0–8	-
Paranoid personality ratings	2.06	2.58	0–12	-	1.65	2.11	0–10	-
Avoidant personality ratings	2.56	3.10	0–12	-	1.83	2.47	0–11	-
Social and occupational functioning	86.0	8.7	55–100	-	85.1	8.26	60–100	-
Global assessment of functioning	84.8	11.0	51–100	-	81.1	11.3	50–100	-
Rosenberg total	22.2	5.21	3–30	.90	22.9	5.28	7–30	.90
Beck depression inventory	5.94	5.46	0–25	.85	6.17	6.80	0–28	.90
Calgary depression scale	1.24	1.87	0–11	-	1.55	2.41	0–11	-

^a^ Coefficient alpha reported for questionnaire measures only.

### Validity of the schizotypy dimensions

Hierarchical linear regressions were computed to examine the variance accounted for by T1 assessments of positive and negative schizotypy in measures of T3 psychopathology, personality, and functioning (Table 2). Positive and negative schizotypy dimension scores were entered simultaneously in the regression models at the first step. In the second step, the T2 measure of the current T3 criterion was entered as a predictor to examine the stability of these measures across measurements. Finally, at the third step, the interaction of both schizotypy dimension with the step 2 measure was entered in order to examine whether positive and negative schizotypy were associated with trait stability or maintenance of symptoms across the assessments. The standardized regression coefficient (β), change in *R*^*2*^, and effect size *f* ^2^ were reported for each predictor in the regressions. Following Cohen [44], *f* ^2^ values above .15 are medium and above .35 are large effect sizes. Given that many of the dependent variables were skewed (especially measures of psychopathology), maximum likelihood estimation and bootstrap procedures (with 2 000 samples) were used.

**Table 2:** Linear regressions of measures of psychosis spectrum, affective dysregulation, self-esteem and functioning.

	Step 1 (df = 1,100)						Step 2 (df = 1,99)			Step 3 (df = 1,97)							
	T1 Positive schizotypy			T1 Negative schizotypy			Criterion T2			Interaction Pos SZ x Criterion T2			Interaction Neg SZ x Criterion T2				
Criterion T3	β	ΔR ^2^	*f* ^2^	β	ΔR ^2^	*f* ^2^	β	ΔR ^2^	*f* ^2^	β	ΔR ^2^	*f* ^2^	β	ΔR ^2^		*f* ^2^	
**Psychosis Spectrum**																	
CAARMS Positive symptoms	.234*	.055	.06	.149	.022	.02	.498*	.227	**.33**	-.024	.000	.00	.096	.007		.01	
CAARMS Negative symptoms	.140	.020	.02	.119	.014	.01	.377***	.121	.14	-.066	.004	.00	.101	.009		.01	
CAARMS Cognitive symptoms	.072	.005	.01	.137	.019	.02	.488***	.232	**.31**	.016	.000	.00	.025	.001		.00	
CAARMS Emotional disturbance	.104	.011	.01	.302**	.091	.10	.210	.038	.04	-.142	.019	.02	.068	.004		.00	
CAARMS Behavioral symptoms	.200	.040	.04	.034	.001	.00	.218*	.043	.05	-.139	.017	.02	-.067	.004		.00	
CAARMS Motor/physical symptoms	.277*	.076	.09	.249*	.062	.07	.313*	.094	.12	.149	.019	.03	.237	.026		.04	
CAARMS General psychopathology	.285**	.081	.09	.067	.004	.00	.538***	.242	**.36**	-.120	.013	.02	.050	.002		.00	
Schizotypal personality	.240*	.058	.07	.273**	.074	.09	.624***	.331	**.62**	.057	.002	.00	.092	.008		.02	
Schizoid personality	.086	.007	.01	.553***	.306	**.45**	.299	.067	.11	-.129	.014	.02	.083	.005		.01	
Paranoid personality	.330**	.109	.13	.219**	.048	.06	.594***	.295	**.54**	.211*	.033	.06	-.109	.010		.02	
Avoidant personality	.294*	.086	.10	.212*	.045	.05	.720***	.418	**.94**	.060	.003	.01	.006	.000		.00	
**Affective dysregulation and self-esteem**																	
Rosenberg total	-.446***	.198	**.26**	-.144	.021	.03	.707***	.360	**.86**	-.058	.002	.00	.031	.001		.00	
Calgary depression scale	.360**	.129	**.15**	-.034	.001	.00	.440***	.180	**.26**	.198	.022	.03	.079	.004		.01	
Beck depression inventory	.238*	.057	.06	.141	.020	.02	.491***	.192	**.26**	-.046	.002	.00	.103		.009		.01
**Functioning**																	
Social and occupational functioning	-.136	.018	.02	-.373**	.139	**.17**	.542***	.249	**.43**	-.099	.009	.02	.290**	.059		.12	
Global assessment of functioning	-.331**	.110	.14	-.287**	.082	.10	.554***	.249	**.45**	-.027	.001	-.02	.133	.015		.03	

*p<0.05

**p<0.01

***p<0.001.

Note 1: A series of linear regressions were computed to examine the variance accounted for by positive and negative schizotypy (T1) in predicting psychopathology, personality and functioning at T3; maximum likelihood estimation and bootstrap procedures (with 2 000 samples) were employed.

Note 2: According to Cohen [44], *f* ^2^ values above .15 are medium and above .35 are large effect sizes.

As hypothesized, the T1 positive schizotypy dimensional score predicted positive (psychotic-like) symptoms, depression, low self-esteem, and general psychopathology at T3. In contrast, T1 negative schizotypy predicted emotional disturbances and schizoid personality ratings at T3. As expected, both positive and negative schizotypy predicted schizotypal, paranoid, and avoidant personality ratings, and global functioning ratings as well as motor/physical symptoms.

**Fig 1:**
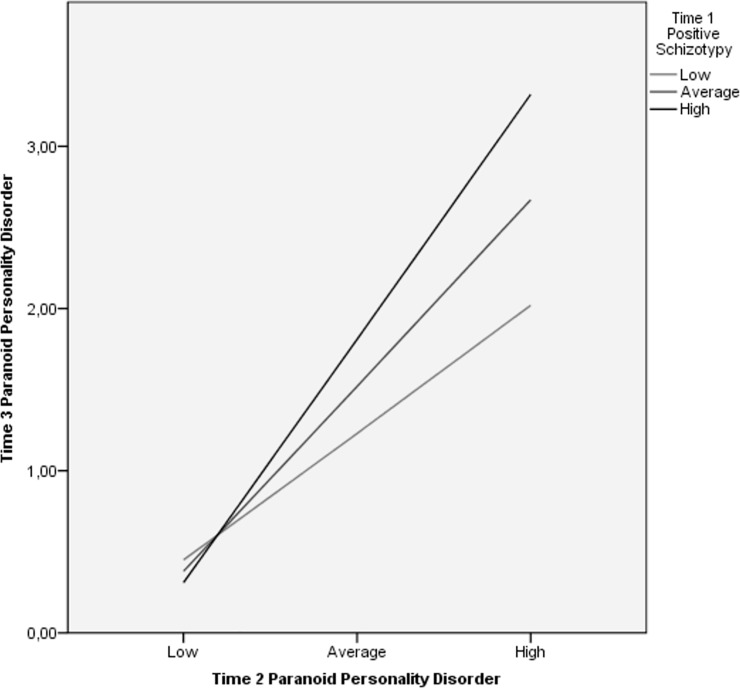
Relationship between T2 and T3 paranoid personality symptoms across levels of T1 positive schizotypy. Relationship between levels of T2 paranoid personality symptoms and T3 paranoid personality symptoms at three levels of T1 positive schizotypy (low, medium, high) as indicated by simple slope analysis.

**Fig 2:**
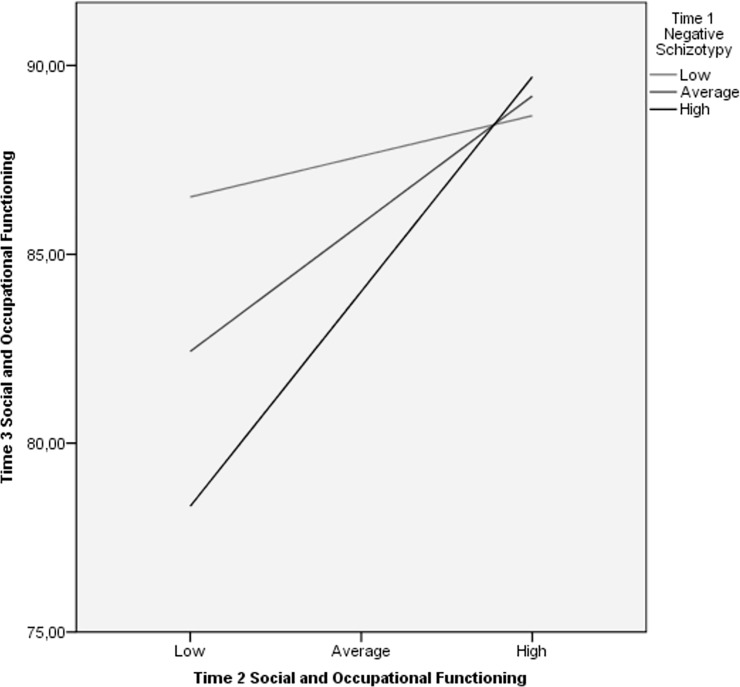
Relationship between T2 and T3 social and occupational functioning across levels of T1 negative schizotypy. Relationship between levels of T2 social and occupational functioning and T3 social occupational functioning at three levels of T1 negative schizotypy (low, medium, high) as indicated by simple slope analysis.

At the second step, T2 measures of symptoms and functioning generally predicted their analogous constructs at T3 over-and-above positive and negative schizotypy. Furthermore, psychosis-spectrum symptom measures generally exhibited lower stability across time in the prediction of T3 analogous constructs than schizophrenia-spectrum personality measures. The interaction of T1 positive and negative schizotypy dimensions with T2 symptom ratings were generally unassociated with measures at T3. However, the interaction of positive schizotypy and T2 paranoid personality ratings predicted T3 paranoid personality traits, whereas negative schizotypy and T2 social and occupational functioning interaction predicted T3 functioning. Simple slope analyses were computed to decompose these interactions. The relationship between T2 and T3 paranoid personality symptoms was significant at all levels of positive schizotypy (Fig 1). However, the relationship strengthened as positive schizotypy increased from low (β = 0.30, *p* < 0.04), to moderate (β = 0.44, *p* < 0.001), to high levels (β = 0.58, *p* < 0.001). T2 social and occupational functioning ratings and the same construct assessed 1.4 years later were significantly related at moderate (β = 0.39, *p* < 0.001) and high (β = 0.65, *p* < 0.001) levels of negative schizotypy, but not low levels (β = 0.12, *p* = 0.5) (Fig 2).

**Fig 3:**
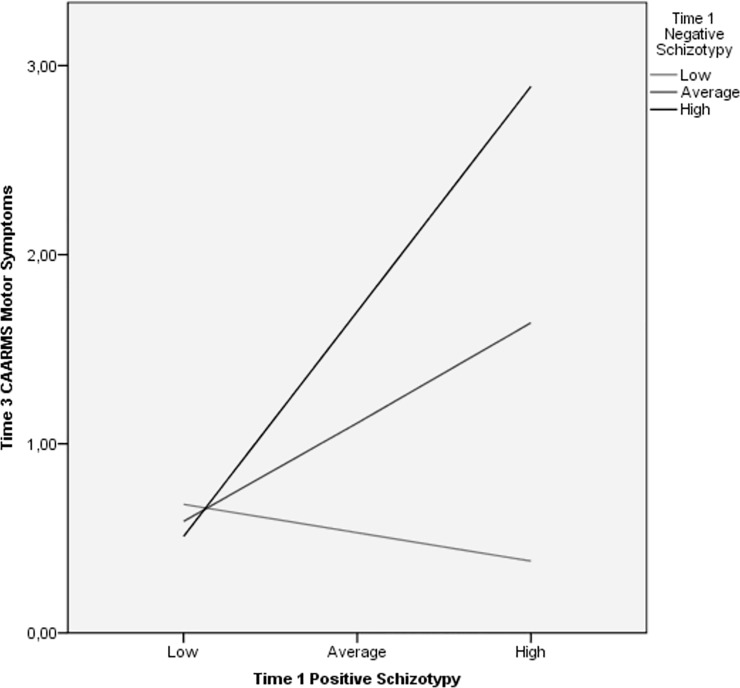
Relationship between T1 positive schizotypy and T3 CAARMS motor/physical symptoms across T1 negative schizotypy levels. Relationship between levels of T1 positive schizotypy and T3 CAARMS motor/physical symptoms at three levels of T1 negative schizotypy (low, medium, high) as indicated by simple slope analysis.

We also examined whether the positive x negative schizotypy interaction terms predicted symptoms and impairment over-and-above the main effects of positive and negative schizotypy. The schizotypy interaction only predicted CAARMS motor/physical symptoms (β = .331, Δ*R*^2^ = .106, *f* ^2^ = .14, *p* < .01) at T3. Simple slope analysis indicated that positive schizotypy and CAARMS motor/physical symptoms were significantly related at moderate (β = 0.49; *p* < 0.05) and high (β = 1.11; *p* < 0.01) levels of negative schizotypy (T1), but not at low levels (β = -0.14; *p* = 0.5) (Fig 3). The lack of significant positive x negative schizotypy interactions is consistent with Kwapil et al. [17] and Barrantes-Vidal et al. [15] who reported additive, but not interactive, effects for positive and negative schizotypy.

We examined the extent to which the schizotypy dimensions predicted prodromal symptom dimensions using SIPS/SOPS ratings at T3 (Table 3). As expected, positive and general prodromal symptoms were predicted by positive schizotypy, whereas negative schizotypy predicted negative prodromal symptoms. Both dimensions predicted total prodromal symptoms.

**Table 3:** Linear regressions of measures of prodromal psychotic SIPS/SOPS symptoms.

	Step 1 (df = 1,100)						Step 2 (df = 1,99)		
	T1 Positive schizotypy			T1 Negative schizotypy			T1 Pos X Neg Schizotypy		
Criterion T3	β	ΔR ^2^	*f* ^2^	β	ΔR ^2^	*f* ^2^	β	ΔR ^2^	*f* ^2^
**SIPS/SOPS**									
Positive	.251*	.063	.07	.173	.030	.03	.233	.052	.06
Negative	.195	.038	.04	.287**	.082	.09	.076	.006	.01
Disorganized	.167	.028	.03	.128	.016	.02	.133	.017	.02
General	.262*	.068	.07	.105	.011	.01	.171	.028	.03
Total	.273*	.074	.09	.232**	.054	.06	.186	.033	.04

*p<0.05

**p<0.01

***p<0.001.

Note 1: A series of linear regressions were computed to examine the variance accounted for by positive and negative schizotypy (T1) in predicting SIPS/SOPS prodromal symptoms and states at T3; maximum likelihood estimation and bootstrap procedures (with 2 000 samples) were employed.

Note 2: According to Cohen [44], *f* ^2^ values above .15 are medium and above .35 are large effect sizes.

Binary logistic regressions were computed to assess the prediction of diagnostic criteria by the schizotypy dimensions. Seven participants assessed at T3 qualified for personality disorder diagnoses at T2: three with Avoidant, two with Schizotypal, and four with Paranoid Personality Disorders (three had more than one disorder). At T3, three of these participants retained schizophrenia-spectrum personality disorders: two with Paranoid and two with Avoidant Personality Disorders (one with two disorders). There were no new cases of personality disorders at T3. Positive schizotypy (OR = 1.97, 95%CI = 0.71–5.51), negative schizotypy (OR = 1.23, 95%CI = 0.39–3.84), and the interaction term (OR = 1.14, 95%CI = 0.40–3.28) failed to predict schizophrenia-spectrum personality disorders at T3. Of the four participants who did not retain their personality disorder diagnosis, three met multiple criteria for personality disorders, but fell short of diagnostic thresholds.

Given that most participants reported sub-diagnostic threshold traits for Cluster A personality disorders, we created an overall Cluster A dimensional score for participants at T2 and T3. This was computed by standardizing and summing the dimensional ratings for schizoid, schizotypal, and paranoid personality disorder at each assessment. We then computed regression analyses predicting the Cluster A rating at T3. Both positive (β = .270, *p* < .01) and negative schizotypy (β = .431, *p* < .001) predicted the ratings, although their interaction term did not (β = .142, *ns*). The Cluster A rating at T2 predicted the rating at T3 (β = .535, *p* < .001); however, neither the interaction of the T2 rating with positive schizotypy (β = .070, *ns*) nor with negative schizotypy (β = .056, *ns*) were significant.

Seven participants who met the CAARMS attenuated psychosis criteria at T2 were reassessed at T3. Of these, three participants continued to meet the attenuated psychosis criteria at T3. There were no new cases meeting CAARMS high-risk criteria at T3. Positive schizotypy (OR = 1.29, 95%CI = 0.44–3.73), negative schizotypy (OR = 1.28, 95%CI = 0.48–3.38), and the schizotypy interaction (OR = 1.23, 95%CI = 0.42–3.62) did not predict CAARMS attenuated psychosis criteria.

Binary logistic regressions were computed in order to examine the prediction of any mental health treatment during the past year at the T3 assessment by schizotypy dimensions assessed at T1. Ten participants reported receiving treatment within the past year. Negative schizotypy (OR = 1.62, *p* < .05), but not positive schizotypy (OR = .91) or the interaction term (OR = 1.10), uniquely predicted mental health treatment at T3.

## Discussion

Multidimensional models of schizotypy provide a useful conceptualization for understanding the underlying developmental vulnerability for schizophrenia-spectrum psychopathology [1, 2]. The psychometric assessment of schizotypy allows us to examine the etiology of schizophrenia-spectrum disorders by identifying individuals with a putative vulnerability for developing such disorders, which should enhance our understanding of etiological factors, inform us about developmental trajectories and risk and protective factors, and potentially provide insights for developing prophylactic interventions. The present study extended our previous findings [15] examining the schizotypy dimensions in a 3-year follow-up of nonclinically ascertained young adults and supported the validity of the two factor structure as distinct dimensions of schizotypy.

Consistent with previous research examining the associations of positive and negative schizotypy with symptoms and impairment [15, 17], both schizotypy dimensions predicted differential associations with psychopathology, personality, and functioning in the present study. Note that the dimensions did not identify additional individuals who had transitioned into schizophrenia-spectrum disorders since the T2 assessment. However, less than 1-1/2 years had passed since that assessment. Furthermore, this is a relatively high functioning sample that has only recently entered into the time of greatest risk for developing schizophrenia-spectrum disorders. Note that the mean age of the sample is only 23 years old, which is younger than Gooding et al.’s [28] and Chapman et al.’s [22] samples at their follow-up.

Negative schizotypy predicted impaired functioning, schizoid and schizotypal personality symptoms and emotional disturbances. The finding that the negative schizotypy dimension did not predict subclinical negative symptoms assessed by the CAARMS is consistent with our previous cross-sectional study [15] and likely reflects the fact that the CAARMS negative symptom rating appears heavily saturated with depression. For example, it correlated moderately with depression (*r* = .45), but only minimally with schizoid symptoms (*r* = .22) and presented a high correlation with CAARMS positive symptoms ratings (*r* = .54). In order to assess negative symptoms in a young adult sample, Kwapil et al. [17] used the Negative Symptom Manual (NSM) [45] to quantify negative symptoms. Findings showed that negative, but not positive, schizotypy had a strong and unique association with NSM ratings. Furthermore, NSM scores were strongly associated with schizoid personality traits, but minimally associated with depression and positive symptoms.

As hypothesized, positive schizotypy predicted psychotic-like symptoms, depression, and low self-esteem, and was related with all schizophrenia-spectrum measures except for schizoid personality. The present study also investigated the association of schizotypy dimensions with depressive symptoms and self-esteem. Our finding that positive, but not negative, schizotypy predicted depression and low self-esteem 3-years later is consistent with our prior cross-sectional study [15] with the present sample. The stronger association of affective symptoms with positive rather than negative schizotypy has been previously reported by cross-sectional (e.g., [46]) and longitudinal studies of non-clinical samples. In the longitudinal study of Chapman et al. [22], participants with elevated scores on the PerAb and MagicId scales reported higher rates of major depressive disorders both at baseline and at 10-year follow-up. In contrast, PhyAnh and SocAnh were not associated with mood disorders at follow-up [22, 26]. These findings suggest that individuals high on positive schizotypy are at greater risk to develop both affective disorders and non-affective psychotic disorders, whereas individuals high on negative schizotypy appear to be at risk especially for schizophrenia-spectrum disorders. Furthermore, evidence indicating that affective experiences are differentially related to positive and negative schizotypy comes from research using ESM. Kwapil et al. [18] reported that negative schizotypy was associated with decreased positive affect in the moment, whereas positive schizotypy was associated with increased negative affect in the moment. Consistent with the latter finding, psychotic-like experiences were related with affective dysregulation in a 10-year longitudinal study in a community sample [47]. It was found that psychotic-like experiences were more likely to have clinical relevance and persist over time with increasing levels of affective dysregulation. Authors suggested that affective dysregulation may causally contribute to the persistence and increasing clinical severity of these experiences through the facilitation of attributions of aberrant salience to abnormal perceptual and cognitive experiences.

The present findings showed that both schizotypy dimensions predicted avoidant personality disorder symptoms, which is consistent with our previous cross-sectional findings [15] as well as with other research conducted with genetic and psychometric high-risk participants showing a link between avoidant personality and liability for schizophrenia. In terms of genetic risk, the UCLA family study [48] demonstrated that avoidant personality disorder occurred more often in individuals with genetic risk for schizophrenia than in control participants, even after controlling for paranoid and schizotypal personality disorders. These authors concluded that such compelling findings suggest that avoidant personality enhances the detection of individuals with vulnerability for schizophrenia and supports the inclusion of avoidant personality as a schizophrenia-spectrum disorder. In fact, Fogelson and colleagues [49] had already indicated that avoidant personality should be included as an additional dimension of schizotypy along with the more traditionally regarded dimensions. In a factor analysis including all schizotypal, schizoid, paranoid, avoidant, and borderline personality disorder traits, avoidant symptoms emerged as a dimension along with positive, negative, disorganized, paranoid, and borderline dimensions. In light of these findings, Gooding et al. [50] reanalyzed data from their 5-year longitudinal study [28] and found that individuals from both SocAnh and PerAb/MagicId groups (especially the former), but not control participants, met criteria for avoidant and Cluster A personality disorders. At the same time, some high-risk participants meeting criteria for avoidant personality disorder did not meet criteria for Cluster A personality disorders. Similarly, Bolinskey et al. [51] found that individuals with elevated schizotypy traits met more criteria for avoidant and Cluster A personality symptoms as compared to control participants, and suggested that avoidant personality disorder may reflect a less severe form of vulnerability for schizophrenia than schizoid personality in which social withdrawal is also associated with conflicting interpersonal feelings. Finally, avoidant symptoms have also been found in schizophrenia patients [52, 53] and in ultra high-risk individuals [54, 55]. Fresán and colleagues [55] reported that avoidant behavior symptoms were more prevalent in ultra high-risk and schizophrenia groups than in control participants and suggested that avoidant personality features may lead to the dysfunctional social interaction observed in both ultra high-risk individuals and schizophrenia patients. Additionally the present results seems to suggest that, on the one hand, avoidant personality is driven by an anxiety component and its association with positive schizotypy and, on the other hand, by the social withdrawal and social disinterest characteristic of negative schizotypy. Social anhedonia (withdrawal/disinterest) and social anxiety have been found to show different patterns of association with affective symptoms, real-life social environment, and schizotypy. Brown et al. [56] reported that social anhedonia was associated with negative schizotypy, whereas social anxiety was associated with positive schizotypy. In a study examining these associations in daily-life by means of ESM, Brown et al. [57] indicated that social anhedonia was associated with decreased positive affect in the moment, reduced desire for social contact, and with preference of solitude. In contrast, social anxiety was found to be associated with increased negative affect in the moment and with the preference to be alone especially when being with people with whom one feels less close to—a situation in which individuals with social anxiety have been found to report the highest level of negative affect. These findings suggested that individuals with high social anxiety desire social contact but feel anxious with non-close others, whereas individuals with social anhedonia are actually not so influenced by the context and present a deficit in affect and disinterest for social interactions.

The present study examined whether baseline measures of schizotypic symptoms and functioning assessed at T2 were associated with the same measures 1.4 years later. As hypothesized, measures generally predicted their analogous ratings at T3, with generally stronger effect sizes for trait than symptoms measures. Unexpectedly, emotional disturbances and schizoid personality ratings at T2 did not significantly predict their equivalent measures at T3. In general, ratings of schizoid personality traits were low and none of the participants qualified for schizoid personality disorders. Nevertheless, negative schizotypy was robustly associated with schizoid traits at T3 (as it had been at T2). The finding that schizoid personality ratings at T2 demonstrated lower stability over time is contrary to our hypothesis and in contrast with the stability of the schizoid psychopathology reported by Lenzenweger [58] in a 4-year longitudinal study. However, consistent with Roberts and Del Vecchio’s [59] meta-analysis, longitudinal research on personality disorders suggested changes in individual personality pathology across time and a degree of flexibility and plasticity rather than fixed stability [60–63].

The findings that schizophrenia-spectrum symptoms predicted their equivalent measure across time with large effect sizes provides further evidence for the stability and the persistence of schizophrenia-spectrum characteristics for those participants who reported high levels of symptoms 1.4 years before the T3 assessment. Hanssen et al. [64] reported that subclinical psychotic experiences in the general population are 100 times greater than the incidence of psychotic diagnoses. In the same line, the epidemiological study of Werbeloff et al. [65] showed that 20–22% of the population reported negative symptoms and of these only a few reported psychiatric clinical diagnoses. This is consistent with previous studies in the general population that reported the presence of subclinical psychotic symptoms is greater than the incidence of psychotic diagnosis [64, 65]. Moreover, De Loore et al. [66] reported that 5% of 1912 adolescents reported auditory hallucinations and these symptoms were persistent in one-third of them. Thus, psychometric study of schizotypy provides a valid method to identify and study developmental trajectories of schizophrenia-spectrum psychopathology in a longitudinal framework.

In general, the association of symptom, trait, and impairment ratings from T2 to T3 did not vary as a function of baseline levels of positive or negative schizotypy at T1. Based upon the main effects for positive and negative schizotypy, this suggests that people high in positive and negative schizotypy tended to report higher levels of symptom, traits, and impairment at T2, which were maintained at T3, whereas people lower in schizotypy tended to have lower scores on interview measures that maintained across the two assessments. Nevertheless, there were two significant interactions. High levels of positive schizotypy at baseline predicted a stronger association of paranoid personality ratings across assessments. Similarly, high levels of negative schizotypy at baseline predicted a stronger association of social impairment.

The present findings provide further evidence of the predictive validity of positive and negative schizotypy dimensions and are consistent with our previous cross-sectional findings [15]. The present study is not without limitations. The fact that at T3 we did not attempt to reassess the entire sample of T2 is a limitation; however, we achieved a high reassessment rate (77%) of the identified pool of participants. Note that the lack of an interview assessment at T1 (when psychometric positive and negative schizotypy were assessed) means that we cannot rule out that some participants were already experiencing symptoms and impairment at baseline. This limits our ability to make specific inferences about the developmental timecourse of the symptoms and impairment from T1 to T3, but does not limit our ability to evaluate differential patterns of associations of T1 positive and negative schizotypy with T3 symptoms and impairment. Furthermore, the interpretations of the associations of T2 and T3 symptoms and impairment were not impacted by the presence or absence of symptoms and impairment at T1, nor were the interactions of positive and negative schizotypy with the T2 –T3 relationships. An advantage of the present longitudinal study is that it recruited participants from a nonclinically ascertained sample, which allows us to examine etiological factors without the confounders associated with the disease and to examine the course of participants who do and do not transition into schizophrenia-spectrum disorders [6]. Although at the three-year follow-up none of the participants transitioned into psychotic disorders, this approach should allow us to identify participants who do so at subsequent assessments. This method may ultimately allow us to identify individuals at risk and to develop intervention strategies aimed at decreasing possible risk factors and increasing protective factors (i.e., quality of life, improve affect, social support, etc.) for the development of schizophrenia-spectrum disorders.

## References

[1] Claridge G. Theoretical background and issues In: Claridge G, editor. Schizotypy: Implications for Illness and Health. Oxford University Press; 1997 pp. 3–18.

[2] Kwapil TR, Barrantes-Vidal N. Schizotypy: looking back and moving forward. Schizophr Bull. 2015; 41 Suppl 2: S366–S373. 25548387 10.1093/schbul/sbu186 PMC4373633

[3] Claridge G, Beech T. Fully and quasi-dimensional constructions of schizotypy In: Raine A., Lencz T, Mednick SA, eds. Schizotypal Personality Disorder. Cambridge University Press; 1995 pp. 192–216.

[4] Debbané M, Barrantes-Vidal N. Schizotypy from a developmental perspective. Schizophr Bull. 2015; 41 Suppl 2: S386–S395. 25548385 10.1093/schbul/sbu175 PMC4373627

[5] Barrantes-Vidal N, Grant P, Kwapil TR. The role of schizotypy in the study of the etiology of schizophrenia spectrum disorders. Schizophr Bull. 2015; 41 Suppl 2: S408–S416. 25810055 10.1093/schbul/sbu191 PMC4373635

[6] Lenzenweger MF. Thinking clearly about schizotypy: hewing to the schizophrenia liability core, considering interesting tangents, and avoiding conceptual quicksand. Schizophr Bull. 2015; 41 Suppl 2: S483–S491. 25810061 10.1093/schbul/sbu184 PMC4373631

[7] Vollema MG, van den Bosch RJ. The multidimensionality of schizotypy. Schizophr Bull. 1995; 21: 19–31. 7770738 10.1093/schbul/21.1.19

[8] Fonseca-Pedrero E, Debbané M, Ortuño-Sierra J, Chan RCK, Cicero DC, Zhang LC, et al The structure of schizotypal personality traits: a cross-national study. Psychol Med. 2017; 17: 1–12. 10.1017/S0033291717001829 28712364

[9] Kwapil TR, Gross GM, Silvia PJ, Raulin ML, Barrantes-Vidal N. Development and psychometric properties of the Multidimensional Schizotypy Scale: a new measure for assessing positive, negative, and disorganized schizotypy. Schizophr Res. 2018; 193: 209–217. 10.1016/j.schres.2017.07.001 28735642

[10] Blanchard JJ, Collins LM, Aghevli M, Leung WW, Cohen AS. Social anhedonia and schizotypy in a community sample: the Maryland longitudinal study of schizotypy. Schizophr Bull. 2011; 37: 587–602. 10.1093/schbul/sbp107 19850669 PMC3080671

[11] Kwapil TR, Gross GM, Silvia PJ, Raulin ML, Barrantes-Vidal N. Prediction of psychopathology and functional impairment by positive and negative schizotypy in the Chapmans’ ten-year longitudinal study. J Abnorm Psychol. 2013; 122: 807–815. 10.1037/a0033759 24016018

[12] Miettunen J, Veijola J, Isohanni M, Paunio T, Freimer N, Jääskeläinen E, et al Identifying schizophrenia and other psychoses with psychological scales in the general population. J Nerv Ment Dis. 2011; 199: 230–238. 10.1097/NMD.0b013e3182125d2c 21451346

[13] Barrantes-Vidal N, Lewandowski KE, Kwapil TR. Psychopathology, social adjustment and personality correlates of schizotypy clusters in a large nonclinical sample. Schizophr Res. 2010; 122: 219–225. 10.1016/j.schres.2010.01.006 20138738

[14] Barrantes-Vidal N, Rosa A, Kwapil TR. An examination of neuroticism as a moderating factor in the association of positive and negative schizotypy with psychopathology in a nonclinical sample. Schizophr Res. 2009; 115: 303–309. 10.1016/j.schres.2009.09.021 19822406

[15] Barrantes-Vidal N, Gross GM, Sheinbaum T, Mitjavila M, Ballespí S, Kwapil TR. Positive and negative schizotypy are associated with prodromal and schizophrenia-spectrum symptoms. Schizophr Res. 2013; 145: 50–55. 10.1016/j.schres.2013.01.007 23402694

[16] Horton LE, Barrantes-Vidal N, Silvia PJ, Kwapil TR. Worries about being judged versus being harmed: disentangling the association of social anxiety and paranoia with schizotypy. PLoS One 2014; 9 (6). 10.1371/journal.pone.0096269 24914672 PMC4051642

[17] Kwapil TR, Barrantes-Vidal N, Silvia PJ. The dimensional structure of the Wisconsin Schizotypy Scales: factor identification and construct validity. Schizophr Bull. 2008; 34: 444–457. 10.1093/schbul/sbm098 17768308 PMC2632435

[18] Kwapil TR, Brown LH, Silvia PJ, Myin-Germeys I, Barrantes-Vidal N. The expression of positive and negative schizotypy in daily life: an experience sampling study. Psychol Med. 2012; 42: 2555–2566 10.1017/S0033291712000827 22716971 PMC12815227

[19] Barrantes-Vidal N, Chun CA, Myin-Germeys I, Kwapil TR. Psychometric schizotypy predicts psychotic-like, paranoid, and negative symptoms in daily life. J Abnorm Psychol. 2013; 122: 1077–1087. 10.1037/a0034793 24364610

[20] Chun CA, Barrantes-Vidal N, Sheinbaum T, Kwapil TR. Expression of schizophrenia-spectrum personality traits in daily life. Personal Disord. 2017; 8: 64–74. 10.1037/per0000141 26461045

[21] Debbané M, Eliez S, Badoud D, Conus P, Flückiger R, Schultze-Lutter F. Developing psychosis and its risk states through the lens of schizotypy. Schizophr Bull. 2015; 41 Suppl 2: S396–S407. 25548386 10.1093/schbul/sbu176 PMC4373628

[22] Chapman LJ, Chapman JP, Kwapil TR, Eckblad M, Zinser M. Putatively psychosis-prone subjects 10 years later. J Abnorm Psychol. 1994; 103: 171–183. 8040487 10.1037//0021-843x.103.2.171

[23] Chapman LJ, Chapman JP, Raulin ML. Body-image aberration in schizophrenia. J Abnorm Psychol. 1978; 87: 399–407. 681612 10.1037//0021-843x.87.4.399

[24] Eckblad M, Chapman LJ. Magical ideation as an indicator of schizotypy. J. Consult. Clin. Psychol. 1983; 51: 215–225. 6841765 10.1037//0022-006x.51.2.215

[25] Chapman LJ, Chapman JP, Raulin M. Scales for physical and social anhedonia. J Abnorm Psychol. 1976; 85: 374–382. 956504 10.1037//0021-843x.85.4.374

[26] Kwapil TR. Social anhedonia as a predictor of the development of schizophrenia-spectrum disorders. J Abnorm Psychol. 1998; 107: 558–565. 9830243 10.1037//0021-843x.107.4.558

[27] Eckblad ML, Chapman LJ, Chapman JP, Mishlove M. The Revised Social Anhedonia Scale; 1982 Unpublished test copies available from T.R. Kwapil, UIUC Department of Psychology, Champaign, NC, 61820.

[28] Gooding DC, Tallent KA, Matts CW. Clinical status of at-risk individuals 5 years later: further validation of the psychometric high-risk strategy. J Abnorm Psychol. 2005; 114: 170–175. 10.1037/0021-843X.114.1.170 15709824

[29] Raine A. The SPQ: a scale for the assessment of schizotypal personality based on DSM-III-R criteria. Schizophr Bull. 1991; 170: 555–564. 10.1093/schbul/17.4.555 1805349

[30] Stefanis NC, Hanssen M, Smirnis NK, Avramopoulos DA, Evdokimidis IK, Stefanis CN, et al Evidence that three dimensions of psychosis have a distribution in the general population. Psychol Med. 2002; 32: 347–358. 11866327 10.1017/s0033291701005141

[31] Kwapil TR, Ros-Morente A, Silvia PJ, Barrantes-Vidal N. Factor invariance of psychometric schizotypy in Spanish and American samples. J. Psychopathol. Behav. Assess. 2012; 34: 145–152.

[32] Ros-Morente A, Rodríguez-Hansen G, Vilagrà-Ruiz R, Kwapil TR, Barrantes-Vidal N. Proceso de adaptación al castellano de las Escalas de Vulnerabilidad a las Psicosis de Wisconsin (Adaptation of the Wisconsin scales of psychosis proneness to Spanish). Actas Esp. Psiquiatr. 2010; 38: 33–41. 20931408

[33] Barrantes-Vidal N, Fañanás L, Rosa A, Caparrós B, Riba MD, Obiols JE. Neurocognitive, behavioural, and neurodevelopmental correlates of schizotypy clusters in adolescents from the general population. Schizophr. Res. 2003; 61: 293–302. 12729881 10.1016/s0920-9964(02)00321-3

[34] Chapman LJ, Chapman JP. Infrequency scale for personality measures; 1983 Unpublished scale available from T.R. Kwapil, UIUC Department of Psychology, Champaign, NC, 61820.

[35] American Psychological Association, 1987 Diagnostic and Statistical Manual of Mental Disorders, third edition (Text Revision). Washington Author, American Psychological Association.

[36] Rosenberg M. Society and the Adolescent Self-Image Princeton: Princeton University Press; 1965.

[37] Yung AR, Yuen H, McGorry PD, Phillips LJ, Kelly D, Dell'Olio M, et al Mapping the onset of psychosis: the comprehensive assessment of at-risk mental states. Aust N Z J Psychiatry. 2005; 39: 964–971. 10.1080/j.1440-1614.2005.01714.x 16343296

[38] Miller TJ, McGlashan TH, Rosen JL, Cadenhead K, Cannon T, Ventura J, et al Prodromal assessment with the structured interview for prodromal syndromes and the scale of prodromal symptoms: predictive validity, interrater reliability, and training to reliability. Schizophr Bull. 2003; 29: 703–715. 14989408 10.1093/oxfordjournals.schbul.a007040

[39] First MB, Gibbon M, Spitzer RL, Williams JBW, Benjamin LS. Structured Clinical Interview for DSM-IV Axis II Personality Disorders (SCID-II) Washington: American Psychiatric Press; 1997.

[40] Goldman HH, Skodol AE, Lave TR. Revising Axis V for DSM-IV: a review of measures of social functioning. Am. J. Psychiatry 1992; 149: 1148–1156. 10.1176/ajp.149.9.1148 1386964

[41] American Psychiatric Association, 2000 Diagnostic and Statistical Manual of Mental Disorders, fourth edition (Text Revision). Washington Author, American Psychiatric Association.

[42] Addington D, Addington J, Maticka-Tyndale E, Joyce J. Reliability and validity of a depression rating scale for schizophrenics. Schizophr Res. 1992; 6: 201–208. 1571313 10.1016/0920-9964(92)90003-n

[43] Beck AT, Steer RA, Brown GK. Manual for the Beck Depression Inventory-II. San Antonio: Psychological Corporation; 1996.

[44] Cohen J. A power primer. Psychol Bull. 1992; 112: 155–159. 19565683 10.1037//0033-2909.112.1.155

[45] Kwapil TR, Dickerson LA. Unpublished results. Negative symptom manual. Unpublished interview manual.

[46] Lewandowski KE, Barrantes-Vidal N, Nelson-Gray RO, Clancy C, Kepley HO, Kwapil TR. Anxiety and depression symptoms in psychometrically identified schizotypy. Schizophr Res 2006; 83: 225–235. 10.1016/j.schres.2005.11.024 16448805

[47] van Rossum I, Dominguez MD, Lieb R, Wittchen HU, van Os J. Affective dysregulation and reality distortion: a 10-year prospective study of their association and clinical relevance. Schizophr Bull. 2011; 37: 561–71. 10.1093/schbul/sbp101 19793794 PMC3080695

[48] Fogelson DL, Nuechterlein KH, Asarnow RA, Payne DL, Subotnik KL, Jacobson KC, Neale MC, Kendler KS. Avoidant personality disorder is a separable schizophrenia-spectrum personality disorder even when con- trolling for the presence of paranoid and schizotypal personality disorders: the UCLA family study. Schizophr Res 2007; 91: 192–199. 10.1016/j.schres.2006.12.023 17306508 PMC1904485

[49] Fogelson DL, Nuechterlein KH, Asarnow RF, Payne DL, Subotnik KL, Giannini CA. The factor structure of schizophrenia spectrum personality disorders: signs and symptoms in relatives of psychotic patients from the UCLA family members study. Psychiatry Res. 1999; 87: 137–46. 10579547 10.1016/s0165-1781(99)00086-4

[50] Gooding DC, Tallent KA, Matts CW. Rates of avoidant, schizotypal, schizoid and paranoid personality disorders in psychometric high-risk groups at 5-year follow-up. Schizophr Res 2007; 94: 373–374. 10.1016/j.schres.2007.04.018 17543501 PMC1989688

[51] Bolinskey PK, James AV, Cooper-Bolinskey D, Novi JH, Hunter HK, Hudak DV, Schuder KM, Myers KR, Iati CA, Lenzenweger MF. Revisiting the blurry boundaries of schizophrenia: spectrum disorders in psychometrically identified schizotypes. Psychiatry Res 2015; 225: 335–340. 10.1016/j.psychres.2014.12.015 25555416

[52] Keshavan MS, Duggal HS, Veeragandham G, McLaughlin NM, Montrose DM, Haas GL, Schooler NR. Personality dimensions in first-episode psychoses. Am J Psychiatry. 2005; 162: 102–109. 10.1176/appi.ajp.162.1.102 15625207

[53] Solano J, De Chávez M. Premorbid personality disorders in schizophrenia. Schizophr Res 2000; 44: 137–144. 10.1016/S0920-9964(99)00203-0 10913745

[54] Lee S, Kim K, Park J, Park J, Kim B, Kang J, Lee E, An SK, Kwon JS. Coping strategies and their relationship to psychopathologies in people at ultra high-risk for psychosis and with schizophrenia. J Nerv Ment Dis 2011; 199: 106–110. 10.1097/NMD.0b013e3182083b96 21278539

[55] Fresán A, León-Ortiz P, Robles-García R, Azcárraga M, Guizar D, Reyes-Madrigal F, Tovilla-Zárate C, de la Fuente-Sandoval C. Personality features in ultra-high risk for psychosis: A comparative study with schizophrenia and control subjects using the Temperament and Character Inventory-Revised (TCI-R). ‎J. Psychiatr. Res 2015; 61: 168–173. 10.1016/j.jpsychires.2014.12.013 25554622

[56] Brown LH, Silvia PJ, Myin-Germeys I, Lewandowski KE, Kwapil TR. The Relationship of Social Anxiety and Social Anhedonia to Psychometrically Identified Schizotypy. J Soc Clin Psychol. 2008; 27: 127–149.

[57] Brown LH, Silvia PJ, Myin-Germeys I, Kwapil TR. When the need to belong goes wrong: The expression of social anhedonia and social anxiety in daily life. Psychol. Sci. 2007; 18: 778–782. 10.1111/j.1467-9280.2007.01978.x 17760772

[58] Lenzenweger MF. A source, a cascade, a schizoid: a heuristic proposal from the Longitudinal Study of Personality Disorders. Dev. Psychopathol. 2010; 22: 867–881. 10.1017/S0954579410000519 20883587

[59] Roberts BW, DelVecchio WF. The rank-order consistency of personality traits from childhood to old age: a quantitative review of longitudinal studies. Psychol Bull. 2000; 126: 3–25. 10668348 10.1037/0033-2909.126.1.3

[60] Grilo CM, Sanislow CA, Gunderson JG, Pagano ME, Yen S, Zanarini MC, et al Two-year stability and change of schizotypal, borderline, avoidant, and obsessive-compulsive personality disorders. J Consult Clin Psychol. 2004; 72: 767–75. 10.1037/0022-006X.72.5.767 15482035 PMC3289406

[61] Johnson JG, Cohen P, Kasen S, Skodol AE, Hamagami F, Brook JS. Age-related change in personality disorder trait levels between early adolescence and adulthood: a community-based longitudinal investigation. Acta Psychiatr Scand. 2000; 102: 265–275. 11089726 10.1034/j.1600-0447.2000.102004265.x

[62] Lenzenweger MF, Willett JB. Does change in temperament predict change in schizoid personality disorder? A methodological framework and illustration from the Longitudinal Study of Personality Disorders. Dev. Psychopathol. 2009; 21: 1211–1231. 10.1017/S0954579409990125 19825265

[63] Shea M, Stout R, Gunderson J, Morey L, Grilo C, McGlashan T, et al Short-term diagnostic stability of schizotypal, borderline, avoidant, and obsessive–compulsive personality disorders. Am J Psychiatry. 2002; 159: 2036–2041. 10.1176/appi.ajp.159.12.2036 12450953

[64] Hanssen M, Bak M, Bijl R, Vollebergh WAM, van Os J. The incidence and outcome of subclinical psychotic experiences in the general population. Br J Clin Psychol. 2005; 44: 181–191. 10.1348/014466505X29611 16004653

[65] Werbeloff N, Dohrenwend BP, Yoffe R, van Os J, Davidson M, Weiser M. The association between negative symptoms, psychotic experiences and later schizophrenia: a population-based longitudinal study. PLoS One 2015; 10 (3). 10.1371/journal.pone.011985 PMC4351950 25748557

[66] De Loore E, Gunther N, Drukker M, Feron F, Sabbe B, Deboutte D, et al Persistence and outcome of auditory hallucinations in adolescence: a longitudinal general population study of 1800 individuals. Schizophr Res. 2011; 127: 252–256. 10.1016/j.schres.2011.01.015 21315559

